# Anti-Liver Fibrosis Activity and the Potential Mode of Action of Ruangan Granules: Integrated Network Pharmacology and Metabolomics

**DOI:** 10.3389/fphar.2021.754807

**Published:** 2022-01-14

**Authors:** Xiaofei Shang, Huixin Yuan, Lixia Dai, Yang Liu, Jian He, Huan Chen, Hongyan Li, Xiuhui Li

**Affiliations:** ^1^ Beijing YouAn Hospital, Capital Medical University, Beijing, China; ^2^ Lanzhou Institute of Husbandry and Pharmaceutical Sciences, Chinese Academy of Agricultural Sciences, Lanzhou, China

**Keywords:** ruangan granules, liver fibrosis, network pharmacology, metabolomics, mode of action

## Abstract

Ruangan granules (RGGs) have been used to treat liver fibrosis with good clinical efficacy for many years. However, the potential mechanism of action of RGGs against liver fibrosis is still unclear. In this study, we evaluated the quality and safety of this preparation and aimed to explore the anti-liver fibrosis activity and potential mode of action of RGGs using network pharmacology and metabolomics. The results showed that RGGs contained abundant ferulic acid, salvianolic acid B and paeoniflorin, and at the given contents and doses, RGGs were safe and presented anti-liver fibrosis activity. They presented anti-liver fibrosis activity by improving liver function (ALT and AST, *p* < 0.01) and pathology and decreasing fibrosis markers in the serum of rats caused by CCl_4_, including HA, LN, PC III, HYP, CoII-V, and α-SMA, and the oxidant stress and inflammatory response were also alleviated in a dose-dependent manner, especially for high-dose RGGs (*p < 0.01*). Further studies showed that RGGs inhibited the activation of the PI3K-Akt signaling pathway in rats induced by CCl_4_, regulated pyrimidine metabolism, improved oxidative stress and the inflammatory response by regulating mitochondrial morphology, and alleviated liver fibrosis. Luteolin, quercetin, morin and kaempferol were active compounds and presented the cytotoxicity toward to LX-02 cells. This study provides an overall view of the mechanism underlying the action of RGGs protecting against liver fibrosis.

## Introduction

Hepatic fibrosis is a dynamic pathological process of repairing chronic liver injuries caused by viral hepatitis, alcoholic hepatitis, nonalcoholic steatohepatitis, drugs, metabolic diseases, autoimmune diseases and congenital abnormalities and leads to liver cirrhosis, portal hypertension, hepatocellular carcinoma, and significant morbidity and mortality worldwide (Pinzani et al., 2001; Lee et al., 2015; Li et al., 2016a; Paik et al., 2020). To date, many studies have been conducted to elucidate the mechanism of liver fibrosis and potential therapeutic approaches. Although increasing evidence indicates that the development of liver fibrosis is a reversible progression (Yu et al., 2019), there is still no effective therapy or drugs available for the treatment of liver fibrosis; hence, it is urgent and necessary to find new alternative drugs and strategies for controlling liver fibrosis (Chen et al., 2018).

In China, traditional Chinese medicines (TCMs) have been proven to have significant efficiency in alleviating liver fibrosis and cirrhosis and even reversing progression (Wang et al., 2020a). Many TCM prescriptions have been recommended and approved by the Chinese government and are widely used in the clinic, including Fuzheng Huayu capsules, Biejia Ruangan tablets, Anluo Huaxian pills, Dahuang Zhechong capsules, Danshen injections, and Liuwei Wuling tablets (Wang et al., 2020a; Tao et al., 2014). In addition, some natural products from raw medicines with variable efficacy in liver disease, such as naringin (El-Mihi et al., 2017), gambogic acid (Yu et al., 2019), picroside I (Xiong et al., 2020), and honokiol (Elfeky et al., 2020), were found.

In recent decades, our group found that Ruangan granules (RGGs) demonstrated good clinical efficacy for the treatment of liver fibrosis by invigorating qi, promoting blood circulation, and softening the hardness of patients. RGGs consist of eight raw materials. Among them, carapax of *Trionyx sinensis* Wiegmann (*Biejia*) was used to nourish yin, reduce heat and soften hardness, roots of *Paeonia lactiflora* Pall. (*Chishao*) could clear heat, disperse blood stasis and relieve body pain, roots of *Astragalus membranaceus* Bunge (*Huangqi*) invigorates qi, promotes yang, and strengthen the exterior, rhizome of *Atractylodes macrocephala* Koidz. (*Baizhu*) was applied to invigorate the spleen and qi, roots of *Angelica sinensis* (Oliv.) Diels (*Danggui*) invigorated and promoted the blood circulation of patients, roots and rhizome of *Salvia miltiorrhiza* Bunge (*Dansheng*) was used to promote blood circulation, remove blood stasis and relieve pain, seeds of *Senna tora* (L.) Roxb. (*Juemingzi*) removed heat, improved eyesight, moistened intestines and improved defecation, and *Prunella vulgaris* L. (*Xiakucao*) was used to clear liver, reduce fire, eliminate and eliminate heat and improve defecation (Committee for the Pharmacopoeia of P.R. China 2015). However, the potential mechanism of RGGs against liver fibrosis is still unclear.

As a commonly hepatotoxin, carbon tetrachloride (CCl_4_) is widely employed to induce animals liver fibrosis, and repeated injection results in the accumulation of reactive oxygen species and inflammation cytokines, which in turn to activate hepatic stellate cells (HSCs) and regulate the signaling pathways for promoting liver fibrosis. Especially for Phosphatidylinositol 3 kinase-protein kinase B (PI3K-AKT) signal pathway, which has been considered as major drug target site, and the activation of this pathway in response to growth factors promotes the survival and proliferation of activated HSCs (Kim et al., 2020).

In this study, after determining the contents of active compounds and evaluating acute toxicity *in vivo*, the antifibrotic effect of RGGs on CCl_4_-induced liver fibrosis in rats was evaluated. The potential mechanism of action was investigated by simplified network pharmacology and metabolomics analysis, and active compounds were explored. This study will lay the foundation for the development of Ruangan granules as an alternative strategy or medicine to control and treat liver fibrosis.

## Materials and Methods

### Raw Materials and RGG Preparation

Eight raw materials of Ruangan granules, carapax of *Trionyx sinensis* Wiegmann (Trionycis Carapax, *Biejia*), roots of *Paeonia lactiflora* Pall. (Paeoniae Radix Rubra, *Chishao*), roots of *Astragalus membranaceus* Bunge (Astragali Radix, *Huangqi*), rhizome of *Atractylodes macrocephala* Koidz. (Macrocephalae Rhizoma, *Baizhu*), roots of *Angelica sinensis* (Oliv.) Diels (Angelicae Sinensis Radix, *Danggui*), roots and rhizome of *Salvia miltiorrhiza* Bunge (Salviae Miltiorrhizae Radix et Rhizoma, *Dansheng*), seeds of *Senna tora* (L.) Roxb. (Cassiae Semen, *Juemingzi*), and fruits of *Prunella vulgaris* L. (Prunellae Spica, *Xiakucao*) were purchased from Beijing Tcmages Pharmaceutical Co. (Beijing China) and were authenticated by Prof. Xiu-Hui Li from Beijing Youan Hospital of Capital Medical University. All species were validated using www.theplantlist.org. All voucher specimens with accession numbers 202001-08 were submitted to the Herbarium of Beijing You`an Hospital, Capital Medical University (Beijing, China).

RGGs were prepared according to a previously described method with minor modifications (Gao et al., 2018). Briefly, after combining all raw materials at the given proportions, they were boiled with 12 volumes of distilled water three times for 1 h each, and then, the solutions were combined, filtered and concentrated by a rotary evaporator at 45°C. After adding β-cyclodextrin, the samples were dried and stored at 4°C for further study. One gram of RGGs was equivalent to 9.5 g of crude material.

### Chemicals and Reagents

Rhein (98%), aurantioobtusin (98%), ferulic acid (98%), paeoniflorin (98%), salvianolic acid B (98%), calycosin (98%) and rosmarinic acid (90%) were purchased from Shanghai Yuanye Biochemical Co., Ltd. (Shanghai, China); formic acid, acetic acid, trifluoroacetic acid and phosphoric acid were purchased from Sinopharm Chemical Reagent Co. (Shanghai, China); ammonium acetate (NH_4_AC), ammonium hydroxide (NH_4_OH), and ammonium fluoride (NH_4_F) were purchased from Sigma Aldrich (Darmstadt, Germany); and methanol and acetonitrile (UPLC and MS grade) were purchased from Fisher Scientific (England).

### Quantification of Phytochemicals *via* Ultra-High Performance Liquid Chromatography

The quantification of active compounds of RGGs was performed on a UPLC Agilent technologies apparatus (1290 Infinity II, Agilent, USA), and a C_18_ column (4.6 × 150 mm; 5 μm, Waters, Ireland) was used in the assay.

According to the methods described by the Committee for the Pharmacopoeia of P.R. China (Ch.P., 2010), RGGs were pretreated, and then, the contents of active compounds were determined using UPLC as follows. For the determination of ferulic acid, the solvent system was composed of acetonitrile (85%) and a 0.085% phosphoric acid solution (15%), the flow rate was 0.8 ml/min, the UV absorbance wavelength was set to 316 nm, and the temperature was set to 30°C. For paeoniflorin, the solvent system was composed of methanol (85%) and a 0.05 mol/L potassium dihydrogen phosphate solution (15%), and the UV absorbance wavelength, temperature and flow rate were set to 230 nm, 35°C and 0.6 ml/min, respectively. For salvianolic acid B, the solvent system consisted of methanol (30%), acetonitrile (10%), formic acid (1%) and water (59%), and the UV absorbance wavelength, temperature and flow rate were 286 nm, 35°C and 0.6 ml/min, respectively. For rosmarinic acid, the solvent system consisted of methanol (42%) and a 0.1% trifluoroacetic acid solution (58%), and the UV absorbance wavelength, temperature and flow rate were 330 nm, 35°C and 0.6 ml/min, respectively. For calycosin, the solvent system was composed of acetonitrile (A) and a 0.2% formic acid solution (B), introduced with gradient elution, with 80–60% solution B eluted at 0–20 min and 60% solution B elated at 20–30 min, and the UV absorbance wavelength, temperature and flow rate were 260 nm, 25°C and 0.8 ml/min, respectively. Finally, for rhein and aurantioobtusin, the solvent system consisted of acetonitrile (50%) and 0.1% phosphoric acid (50%), and the UV absorbance wavelength, temperature and flow rate were 284 nm, 30°C and 0.6 ml/min, respectively. The mobile phase was filtered through a 0.45 mm Millipore filter and degassed prior to use. Quantification was based on the retention times and UV spectra of commercial compounds, and the equation of the curve and the coefficient of determination were calculated. Three replicates were performed.

### Acute Toxicity

This test was approved by the Institute Animal Care and Use Committee of Lanzhou Institute of Husbandry and Pharmaceutical Sciences of Chinese Academy of Agricultural Sciences (SYXK-2014-0002). Mice (18–22 g) were obtained from the Lanzhou Veterinary Institute and were randomly divided into groups (*n* = 10). RGGs were administered orally to mice at concentrations of 2000–5,000 mg/kg. Finally, the mice were observed continuously for behavioral changes for the first 4 h and were subsequently observed for mortality for 24 h after administration (Ghosh 2005). Anti-Liver Fibrosis Activity of RGGs *in vitro*

### Animal Experiments

Fifty male Wistar rats with an average age of 8 weeks weighing 180–220 g were purchased from the Lanzhou Veterinary Research Institute (Lanzhou, China). Rats were fed a standardized diet and water ad libitum. Rats were randomly divided into five groups: the control group, model group, high-dose treatment group (H-RGG, 750 mg/kg), medium-dose treatment group (M-RGG, 500 mg/kg) and low-dose treatment group (L-RGG, 250 mg/kg). Each group contained ten animals. Except for the control group (vehicle), all rats were injected intraperitoneally with 50% (v/v) carbon tetrachloride (CCl_4_) diluted in florence oil (2 ml/kg body weight) twice a week for 9 weeks. From the 5th to the 9th week, rats in the treatment groups were orally administered the corresponding RGGs dissolved in distilled water once daily, and the model group was administered the vehicle only. During the test, the behavioral and clinical signs and mortality of rats were observed and recorded, and the body weights were measured each week. At the end of the test, all rats were fasted overnight and then sacrificed for tissue and blood collection. The blood samples were centrifuged at 3,000 × g for 10 min at 4°C to obtain the serum. The livers and spleens were rapidly removed and weighed, and the liver and spleen indexes were calculated (tissue weight/body weight × 100%). A section of each liver was fixed in a 4% formaldehyde solution for HE assay, and others were stored at −80°C before analysis.

### Liver Function and Fibrosis Markers in Serum

The levels of alanine transaminase (ALT) and aspartate transaminase (AST), as liver function markers, and hyaluronic acid (HA), laminin (LN), type III procollagen (PC III), hydroxyproline (HYP) and type IV collagen (ColIV), as fibrosis markers, in serum were determined according to the protocols of commercially available kits (NJJCBIO, China). α-Smooth muscle actin (α-SMA) in serum was evaluated using an ELISA kit (CUSABIO, China).

### Histological Evaluation

Sections of the liver were sliced and fixed in 10% buffered formalin and embedded in paraffin blocks. Then, they were stained with hematoxylin and eosin (H & E) and Masson’s trichrome for routine histopathological examination. The histological features of the livers were evaluated under a light microscope and photographed with a BA200 digital camera (Motic Electric Group Co., Ltd.).

### Measurements of Serum Antioxidant Indexes and Inflammatory Factors

The serum levels of superoxide dismutase (SOD), catalase (CAT), and malondialdehyde (MDA), as antioxidant indexes, and tumor necrosis factor-α (TNF-α) and interleukin-1β (IL-1β), as inflammatory factors, were measured using enzyme-linked immunosorbent assay kits from Nanjingjiancheng Bio (NJJCBIO, China).

### Network Pharmacology

#### Active Ingredient Screening and Prediction for Drug Targets

The chemical compositions from RGGs were retrieved from the TCMSP (https://tcmsp-e.com/) (Ru et al., 2014) or ETCM (http://www.tcmip.cn/ETCM/) (Xu et al., 2019) database and the literature, and compounds with oral bioavailability (OB) ≥ 30% and drug-likeness (DL) ≥ 0.18 were collected for further studies. Then, the names of active compounds were imported into PubChem (https://pubchem.ncbi.nlm.nih.gov/), and the canonical SMILES of each compound was input into SwissTargetPrediction (http://www.swisstargetprediction.ch/) (Daina et al., 2019) to predict targets.

#### Collection of Gene Targets Associated With Liver Fibrosis and Protein-Protein Interaction Network Construction

“Liver fibrosis” was used as a term in the Genecards database (https://www.genecards.org/) (Stelzer et al., 2016) to search for related genes. The intersection between the liver fibrosis-related targets and the predicted targets of RGGs were selected as common targets. Subsequently, these targets were submitted to the STRING 11.0 database (Szklarczyk et al., 2019) (https://string-db.org/), and a PPI network was constructed with a minimum required interaction score greater than 0.9 confidence. The protein information was imported into Cytoscape to construct the protein-protein interaction (PPI) network and merged with the “Compounds-Targets” network.

#### Bioinformatic Analysis

To evaluate the role of targets and the potential mechanism of action of RGGs by bioinformatic annotation, the hub targets were imported into the Enrichr database (https://metascape.org/) (Zhou et al., 2019) to perform functional annotation and enrichment analysis, and gene ontology (GO) terms and KEGG pathways with *p* < 0.05 were obtained.

### PCR Test

The total RNA of liver tissue in rats was extracted using total RNA extraction kit (Qiagen, Germany), and then were reverse transcribed into cDNAs according to the protocol of RT-PCR kit (Qiagen, Germany). PCR primers of *PI3K* gene for the analysis were forward: 5′-CGA​GAG​TAC​GCT​GTA​GGC​TG-32032, reverse: 5′-AGA​AAC​TGG​CCA​ATC​CTC​CG-3′; forward: 5′-GGT​CAC​CTC​TGA​GA-3′, reverse: 5′-CCA​CAC​ACT​CCA​TGC​TGT​CAT-3′ for *Akt*; and forward: 5′-ATT​CAA​TCC​ATA​GCC​CCG​TC′, reverse: 5′-TGC​ATC​ACT​CGT​TCA​TCC​TG-3′ for *mTOR*. Finally, the relative expression level of each gene was calculated by 2^−ΔΔCT^ method. All tests were performed in triplicate.

### Cytotoxicity

The hepatic stellate cell LX-02 cells was cultured in culture medium prepared with 10% FBS and 90% DMEM under a humidified incubator of 5% CO_2_ at 37°C. The cytotoxicities of eight compounds, includes baicalein, morin, albiflorin, hederagenin, paeoniflorin, kaempferol, luteolin and isorhamnetin against LX-02 cells were evaluated. Briefly, LX-02 cells at a density of 1 × 10^5^ cells/well (100 μL) were incubated in 96-well plates for 24 h, and then 10 μL of compounds (50–250 μg/ml) was added to each well and incubated again for 1 h. TGF-β (5 ng/ml) was added to each well for 24 h to activate LX-02 cells. Finally, after adding CCK-8 agent (10 μL) and culturing continuously for 30 min, and the absorbance was measured at 450 nm.

### Molecular Docking

To prove the result of network pharmacology, the molecular docking between three identified targets and three active compounds was performed using the DOCK 6.9 software in Yinfo Cloud Platform (http://cloud.yinfotek.com/). The grid score (kcal/mol), and internal energy (kcal/mol) based on the best pose was calculated.

### Metabolomics

#### LC-MS/MS Analysis

Serum was mixed with 100 μL aliquots and 400 μL of cold methanol/acetonitrile (1:1, v/v) to remove proteins. After centrifugation for 15 min (14,000 × *g*) at 4°C, the supernatant was dried and redissolved in acetonitrile/water (1:1, v/v) for LC-MS/MS analysis, which was performed using a UHPLC instrument (1290 Infinity LC, Agilent Technologies) coupled to a quadrupole time-of-flight instrument (AB Sciex Triple TOF 6600), and a 2.1 × 100 mm ACQUIY UPLC HSS T3 1.8 µm column (Waters, Ireland)was used. The mobile phase was composed of a 0.1% formic acid solution (A) and acetonitrile with 0.1% formic acid (B) for positive mode and a 0.5 mM ammonium fluoride solution (A) and acetonitrile (B) for negative mode. The gradient was 1% B for 1.5 min, which was increased to 99% in 11.5 min, where it was maintained for 3.5 min; then, it was reduced to 1% in 0.1 min, and a 3.4 min re-equilibration period was employed. The flow rate was 0.3 ml/min, and the column temperature was 25°C. A 2 µL aliquot of each sample was injected.

For the ESI source conditions, the ion source gas 1 and ion source gas 2 were set as 60, the curtain gas was 30, the source temperature was 600°C, and the ion spray voltage was ±5500 V. In MS acquisition, the instrument was set to acquire over the m/z range of 60–1,000 Da, and the accumulation time was set at 0.20 s/spectra. In MS/MS acquisition, the instrument was set to the m/z range of 25–1,000 Da, and the accumulation time was set at 0.05 s/spectra. The collision energy was fixed at 35 V with ±15 eV; the declustering potential was 60 V (+) and −60 V (-); and isotopes within 4 Da were excluded.

#### Data Processing

The raw MS data (wiff.scan files) were converted to MzXML files using ProteoWizard MSConvert. The intensity of each ion was normalized by using an internal standard, and the output was exported into SIMCA software for principal component analysis (PCA) and orthogonal partial least-squares discriminant analysis (OPLS-DA). Putative markers were selected in accordance with variable importance in projection values (VIP >1) and independent t-tests (*p* < 0.05). Potential markers were tentatively identified using METLIN (http://metlin.scripps.edu) databases and analyzed using MetaboAnalyst5.0 (https://www.metaboanalyst.ca/).

### Statistical Analysis

All statistical analyses were performed using GraphPad Prism version 8.0.1. The data were analyzed using ordinary one-way ANOVA with Tukey’s test when the data involved three or more groups.

## Results

### The Contents of Active Compounds in RGGs

To control the quality of raw materials in RGGs, the contents of seven compounds were quantified by UPLC analysis (Supplementary Figure S1). As shown in Table 1, the contents were 7.70 mg/g for ferulic acid from *Angelica sinensis* (Oliv.) Diels, 1.43 mg/g for paeoniflorin from *Paeonia lactiflora* Pall., 2.00 mg/g for salvianolic acid B from *Salvia miltiorrhiza* Bunge, 1.71 mg/g for rosmarinic acid from *Prunella vulgaris* L., 0.03 mg/g for calycosin from *Astragalus membranaceus* Bunge, and 1.33 mg/g for rhein and 0.57 mg/g for aurantioobtusin from *Senna tora* (L.) Roxb. At the given contents of active compounds, RGGs had a good effect in the subsequent assay.

**TABLE 1:** Contents of active compounds from RGGs.

Compounds	Retention time (min)	Contents (mg/g^a^)	Regression	R^2^	Species
Ferulic acid	14.7	7.70	Y = 141.89X-11.568	0.998	*Angelica sinensis* (Oliv.) Diels
Paeoniflorin	7.5	1.43	Y = 3291.6X-255.4	0.998	*Paeonia lactiflora* Pall.
Salvianolic acid B	9.5	2.00	Y = 1718.7X-13.454	0.999	*Salvia miltiorrhiza* Bunge
Rosmarinic acid	11.9	1.71	Y = 4348.7X-170.76	0.999	*Prunella vulgaris* L.
Calycosin	4.56	0.03	Y = 32.139X+8.551	0.998	*Astragalus membranaceus* Bunge
Rhein	4.31	1.33	Y = 4936.1X-6.2489	0.999	*Senna tora* (L.) Roxb.
Aurantioobtusin	26.96	0.57	Y = 2918.6X-10.524	0.999	*Senna tora* (L.) Roxb.

a mg compound/g RGGs.

### Toxicity

To ensure the safety of RGGs, we evaluated acute toxicity in mice. After oral administration of RGGs (5,000 mg/kg) to mice, no mice died or exhibited any acute behavior, and the LD_50_ was determined to be more than 5,000 mg/kg. This result suggested that at the concentration employed in this study, the RGGs were safe.

### RGG Protection Against CCl_4_-Induced Liver Fibrosis

**FIGURE 1:**
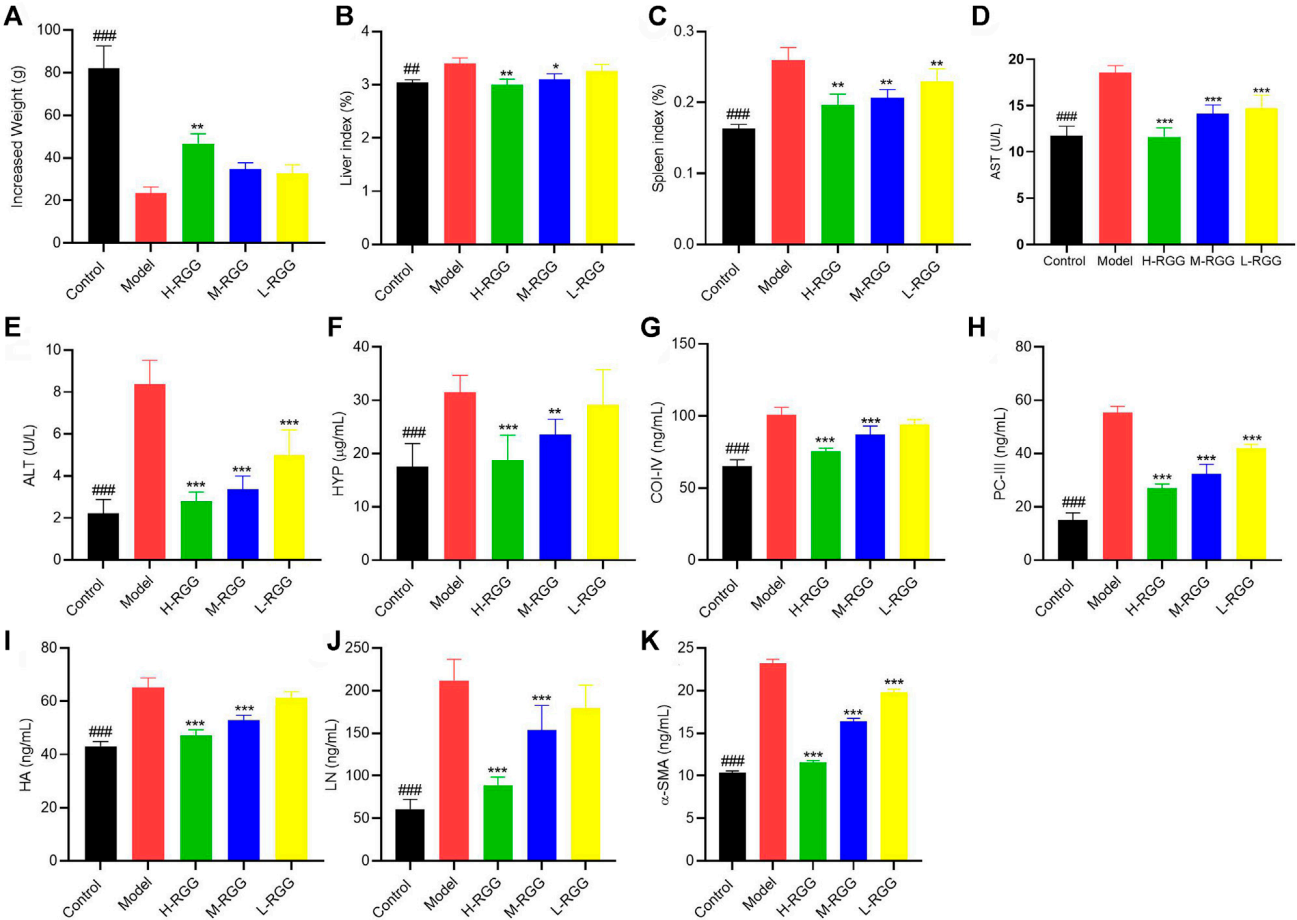
RGG presented anti-liver fibrosis activity *in vivo* [**(A)**, increased weight; **(B)** liver index; **(C)** spleen index; and **(D)** AST activity; **(E)** ALT activity; **(F)** HYP content; **(G)** COI-IV content; **(H)** PC-III content; **(I)** HA content; **(J)** LN content and **(K)** α-SMA content in the serum of rats] ^###^ *p < 0.001* compared with the model for the control group; *p < 0.05, **p < 0.01, and ***p < 0.001 compared with the model group for the RGG-treated groups.

As a chemical toxin, CCl_4_ causes liver fibrosis and induces fatigue, depression, lack of exercise, unsociable behavior, poor appetite, abdominal distension and skin bruising in rats (Nazhvani et al., 2020). However, after RGG treatment, the behavior and status of rats became normal. At the end of the test, the average increased weight of H-RGG (46.67 g) was significantly higher than that of the model group (23.33 g) (*p < 0.01*). The average increased weight of the control group was 82.00 g (Figure 1A). At the same time, the weights of the liver and spleen were not remarkably enhanced, and the liver index and spleen index were all lower than those of the model group, especially for the H-RGG group (*p < 0.01*) (Figures 1B,C). This result indicated that RGGs may decrease the production of fibrotic proteins and alleviate swollen liver and spleen in a dose-dependent manner.

### RGG-Induced Improvement in Liver Function and Liver Fibrosis in Rats Induced by CCl_4_

Subsequently, the levels of ALT and AST, as liver function markers, were evaluated. Figures 1D,E shows that after injecting CCl_4_ into rats for 9 weeks, the levels of ALT (8.39 U/L) and AST (18.53 U/L) in serum were significantly increased compared with those in the control group (2.23 U/L and 11.78 U/L, respectively) (*p < 0.001*). However, RGG treatment remarkably decreased the increased ALT and AST levels in serum in a dose-dependent manner (*p < 0.001*). This result indicated that RGGs significantly improved the liver function of rats induced by CCl_4_.

In addition, we found that RGGs alleviated liver fibrosis by decreasing the levels of fibrosis markers in the serum of rats caused by CCl_4_, including HA, LN, PC III, HYP and CoII-V, especially at high and medium doses (*p < 0.001*) (Figures 1F–J). Finally, the value of α-SMA in serum was determined, as it is one of the most well-established hallmarks of myofibroblast differentiation (Wang et al., 2019). The results showed that compared with the model group (23.18 ng/ml), RGG treatment significantly inhibited the expression of α-SMA in a dose-dependent manner (*p < 0.001*), and the contents of α-SMA in serum were 11.58 ng/ml for H-RGG, 16.39 ng/ml for M-RGG and 19.81 ng/ml for L-RGG. The serum level of α-SMA in the control group was 10.36 ng/ml (Figure 1K).

### RGG-Induced Alleviation of the Histological Change in Rats Induced by CCl_4_

**FIGURE 2:**
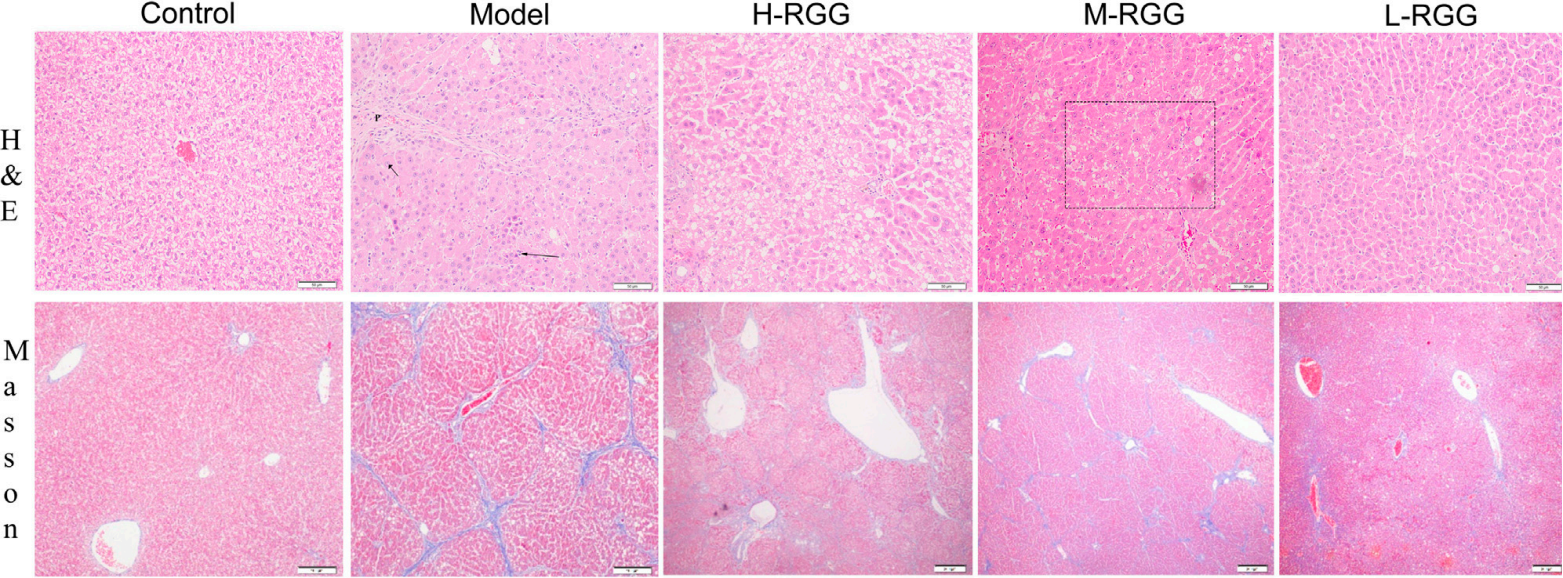
RGG alleviated histological changes in rats induced by CCl_4_, as shown by H&E staining and Masson staining.

To prove the anti-liver fibrosis effect of RGGs *in vivo*, the histological properties of the liver were evaluated using HE staining and Masson staining. Figure 2 shows that compared with the normal morphology of liver tissue in the control group, the liver tissue in the model group exhibited fibrosis (black arrow) and a dense inflammatory infiltrate in H&E staining; however, after treatment with RGGs, the liver morphology of hepatocytes became normal in a dose-dependent manner, especially for H-RGG. Masson staining analysis demonstrated that fibrous septa appeared and that collagen was deposited in the CCl_4_-induced liver fibrosis group, and the histological properties indicated that rats had serious liver fibrosis or early-stage cirrhosis. Fortunately, after RGG treatment, some fibrous septa disappeared, and collagen was alleviated compared with the model group. Taken together, RGG had a protective effect on liver fibrosis and could alleviate or even reverse the histological change in rats induced by CCl_4_.

### RGG-Induced Improvement in the Oxidant Stress and Inflammatory Response of Rats Induced by CCl_4_

**FIGURE 3:**
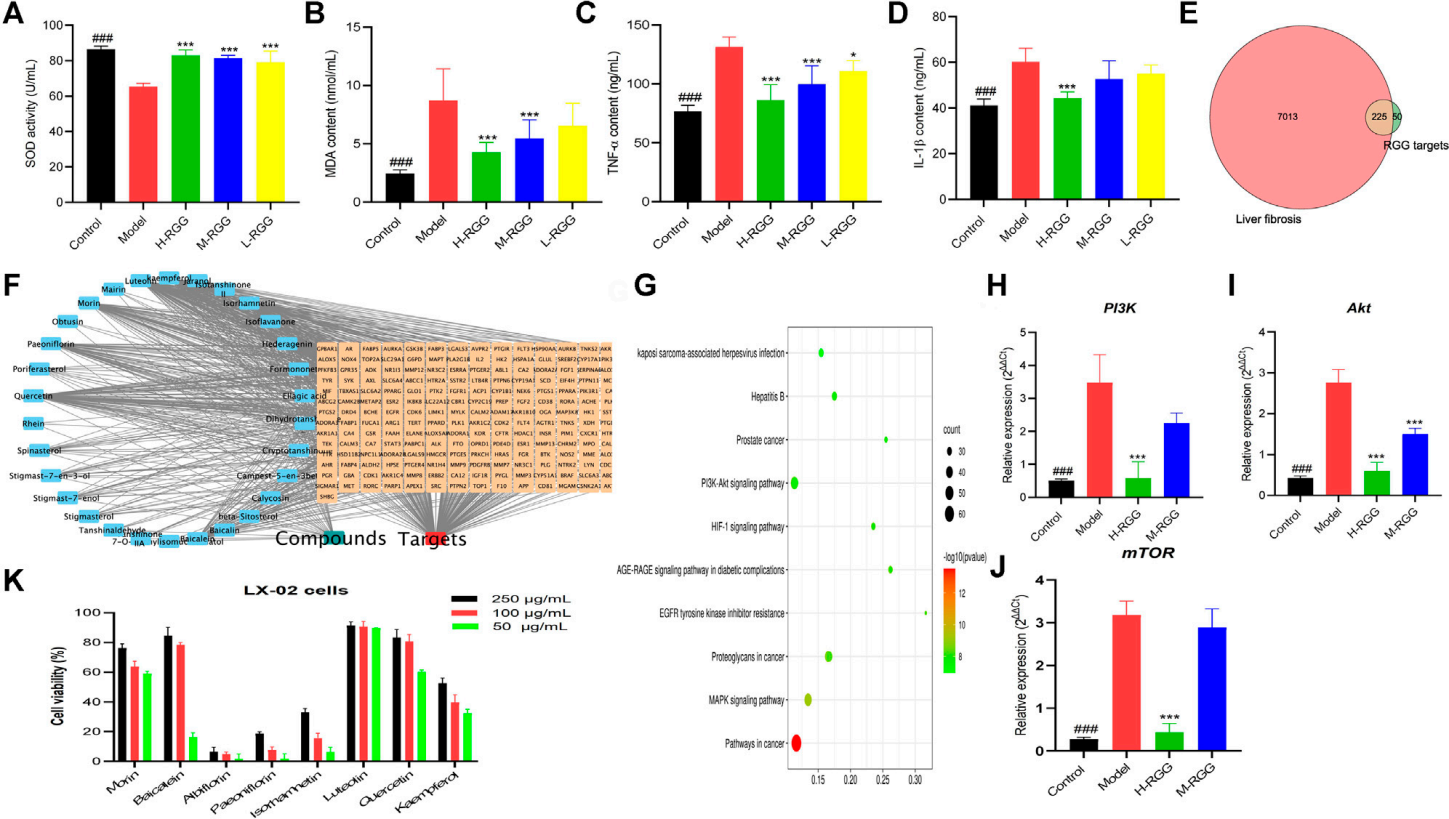
RGG alleviated oxidative stress [**(A)** SOD activity; **(B)** MDA content] and the inflammatory response [**(C)** TNF-α content and **(D)** IL-1β content]. The common targets between liver fibrosis and RGGs **(E)** in network pharmacology, the compound-target network **(F)** and the KEGG pathway **(G)**, the gene expression of *PI3K* **(H)**, *AKT* **(I)** and *mTOR* **(J)** in PCR test, and the cell viability of eight compounds **(K)** ^###^ *p < 0.001* compared with the model for the control group; *p < 0.05, and ***p < 0.001 compared with the model group for the RGG-treated groups.

Along with the development of liver fibrosis in rats induced by CCl_4_, oxidant and inflammatory cytokines were generated and produced. Hence, the levels of SOD and MDA were determined as oxidant indexes, and TNF-α and IL-1β were determined as inflammatory cytokines to evaluate the effects of RGGs on oxidative stress and the inflammatory response of rats induced by CCl_4_. The results showed that compared with the model group (65.40 U/mL), RGGs significantly enhanced SOD activity in a dose-dependent manner, and the activities were 83.04 U/mL for H-RGG, 81.42 U/mL for M-RGG, and 79.23 U/mL for L-RGG (*p < 0.001*). The SOD activity of the control group was 86.50 U/mL. RGGs also decreased the MDA value in serum compared with the model group in a dose-dependent manner (Figures 3A,B). These results indicated that RGGs could alleviate the oxidative stress induced by CCl_4_ in rats.

In addition, as shown in Figures 3C,D, RGGs significantly reduced the levels of TNF-α, with contents of 86.29 ng/ml for H-RGG (*p < 0.001*), 99.94 ng/ml for M-RGG (*p < 0.001*), and 111.03 ng/ml for L-RGG (*p < 0.05*). The TNF-α levels in the control group and model group were 76.65 ng/ml and 131.50 ng/ml, respectively. After RGG treatment, the levels of IL-1β in serum were also decreased compared with those in the model group, especially for H-RGG (*p < 0.001*). These results indicated that RGG treatment improved the inflammatory response of rats induced by CCl_4_.

### Network Pharmacology

In this study, 134 compounds with good OB and DL were collected from RGGs; however, only 51 compounds had 275 unduplicated potential targets, and the probability was more than 0.12. Meanwhile, 7,238 targets related to liver fibrosis were found after searching the Genecards database, and 225 common targets between RGGs and liver fibrosis were obtained for further assays (Figure 3E).

**FIGURE 4:**
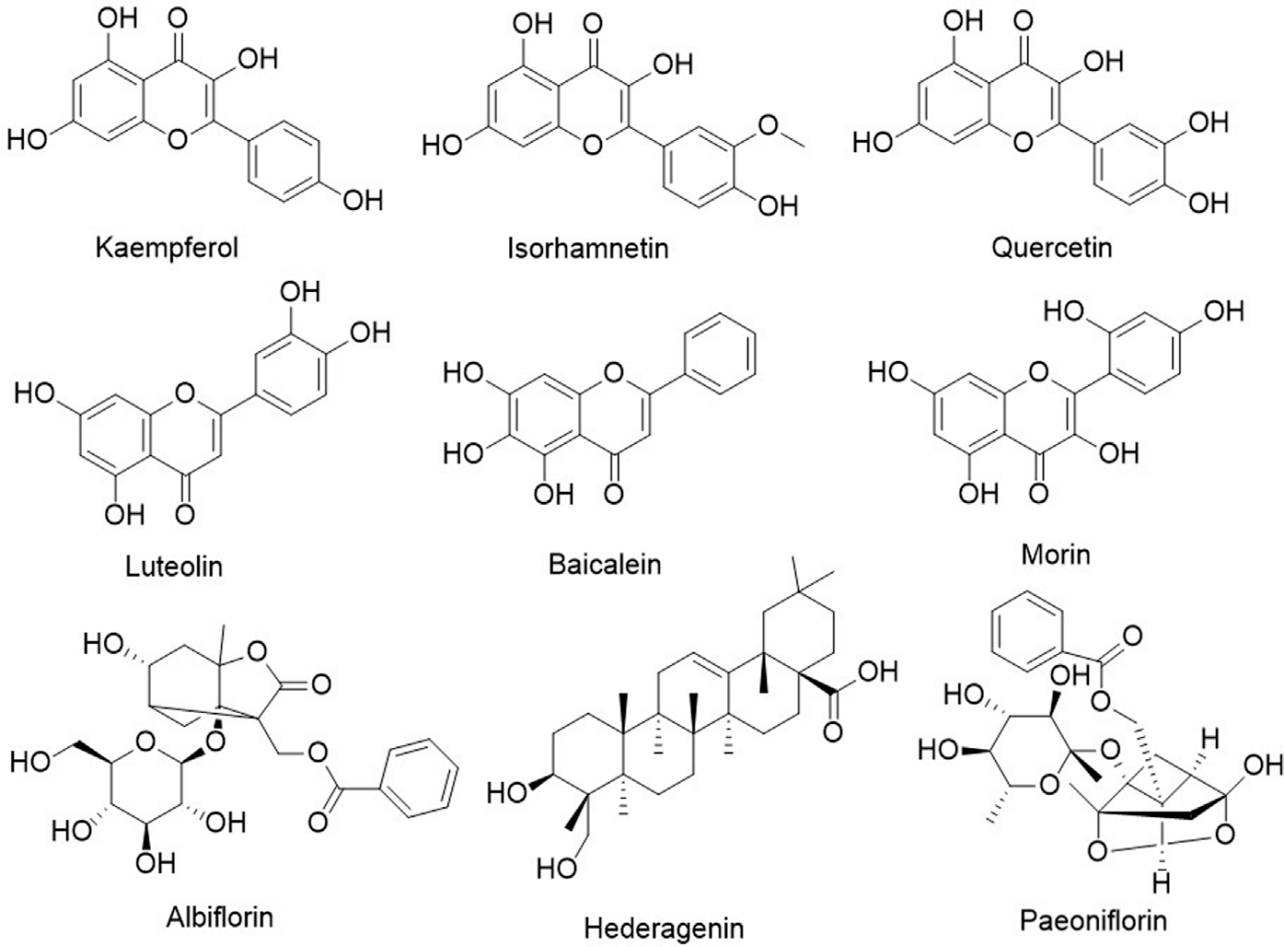
Chemical structures of the active compounds in RGGs.

Then, a PPI network was constructed using the STRING 11.0 database with a minimum required interaction score greater than 0.9 for the highest confidence (Supplementary Figure S2). The numbers of nodes and edges were 217 and 585, respectively, the average node degree was 5.39, and the *p* value of PPI enrichment was less than 1.0E-16. In PPIs, we noticed that SRC, PIK3R1, STAT3, AKT1, HRAS, HSP90AA1 and the related targets had more edges and nodes. To obtain more valuable information about the potential mechanism of action of RGGs against liver fibrosis and its active compounds, a compound-target network was established and constructed. The results showed that as potential active compounds in RGGs, kaempferol, isorhamnetin, quercetin, and luteolin could act on more than 60 targets related to liver fibrosis, followed by baicalein, morin, albiflorin, hederagenin, and paeoniflorin, which had effects on the targets between 40 and 59 (Figure 4). Ellagic acid presented multitarget activities. In addition, among all common targets for RGGs and liver fibrosis, ESR1, ESR2, AR, ACHE, CYP19A1, and AKR1B1 have a high degree of interaction with other targets and some active compounds (Figure 3F).

GO analysis showed that these genes were enriched in the regulation of the MAPK cascade, positive regulation of transferase activity, positive regulation of kinase activity and other biological processes and participated in cellular components, such as receptor complexes, membrane complexes, membrane rafts, and membrane microdomains; these genes were involved in kinase activity, kinase binding, phosphotransferase activity and alcohol groups as receptors, and others were involved in molecular functions (Supplementary Figure S3).

Finally, KEGG pathway analysis showed that the potential targets of active compounds in RGGs were mainly enriched in pathways in cancer, the PI3K-AKT signaling pathway, the MAPK signaling pathway, and others (Figure 3G). These results indicated that RGGs presented anti-liver fibrosis activity by regulating the PI3K-AKT and MAPK pathways, and further studies were performed to prove this hypothesis.

### PCR Test

For proving the result of network pharmacology, qRT-PCR assay was performed the expressions of three genes related to PI3K-Akt pathway. As shown in Figures 3H–J, after RGG treatment the expressions of three genes (*PI3K, Akt* and *mTOR*) were down-regulated with the dose-dependent manner compared with the model group, especially for high dose group (*p < 0.01*). This results indicated that RGG could inhibited the activation of PI3K-Akt pathway induced by CCl_4_ to block the progress of liver fibrosis.

### Cytotoxicity

From Figure 3K, we can see that most of compounds presented the inhibitory effect on LX-02 cells induced by TGF with a concentration-dependent manner, and luteolin, quercetin and morin showed the strongest cytotoxicities with the inhibition rates of 89.78, 60.28 and 59.21% at the concentration of 50 μg/ml, kaempferol also has the moderate activity. However, the cytotoxicity of albiflorin, hederagenin and paeoniflorin were weak. This result indicated that RGG may played the anti-liver fibrosis activity through multiple pathways and multiple targets, and luteolin, quercetin, morin and kaempferol were active compounds.

### Molecular Docking

**FIGURE 5:**
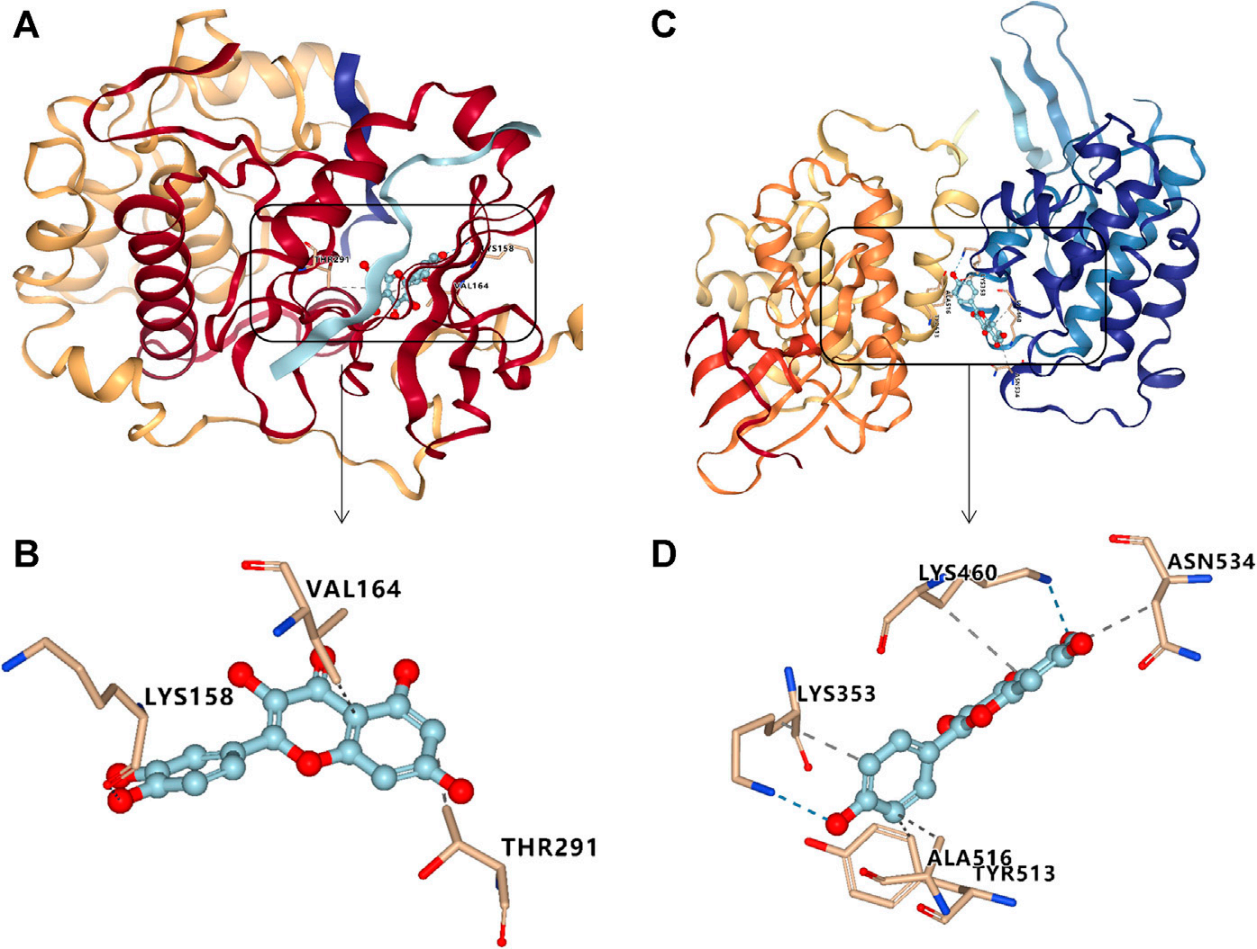
Molecular docking results for targets with major compounds: quercetin- AKT1 **(A, B)**, and kaempferol-SRC **(C, D)**.

In this study, we made the molecular docking between the target SRC (PDB 4U5J) and active compound kaempferol, and AKT1 (3QKL) and quercetin to prove their affinity and find the binding core. Result showed that quercetin has the high affinity to the target AKT1 with the grid score of −57.76 kcal/mol, and the internal energy was 6.23 kcal/mol. The further study showed that it could bind to Lys (158), Val (164) and (Thr 291) of SRC (Figures 5A,B). Kaempferol also could bind to Lys (353, 463), Thr (513), Ala (516) and Asn (534) of SRC with the high affinity, the grid score and the internal energy of -54.30 kcal/mol and 5.50 kcal/mol, respectively (Figures 5C,D).

### Metabolomics

**FIGURE 6:**
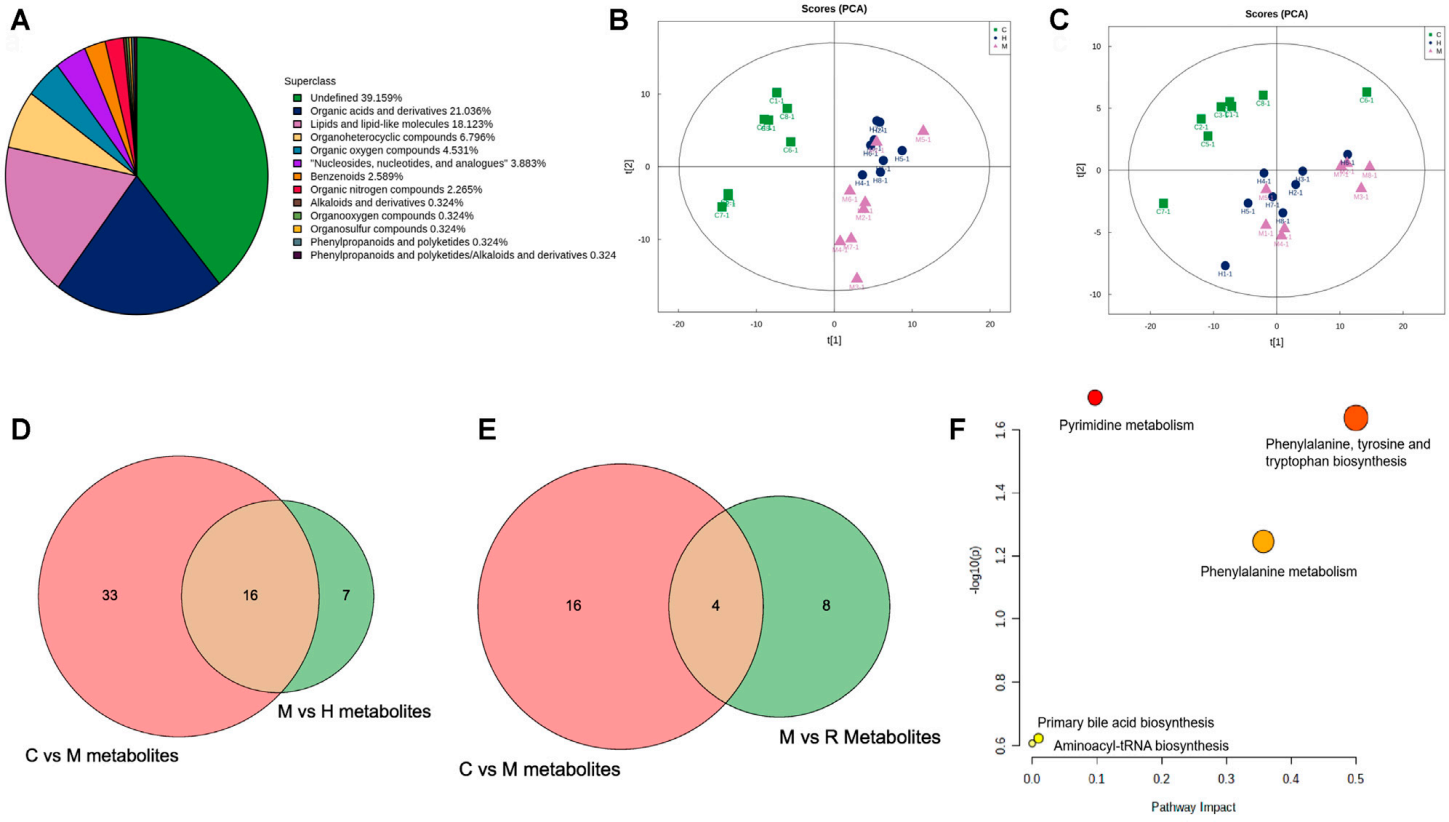
Class pie chart of the identified metabolites in the liver **(A)**, the PCA scores obtained in multivariate statistical analysis for negative ion mode **(B)** and for positive ion mode **(C)**, the common metabolites of the control model vs. model and the model vs. H-RGG for negative ion mode **(D)** and for positive ion mode **(E)**, and the KEGG pathway for metabolites **(F)**.

**FIGURE 7:**
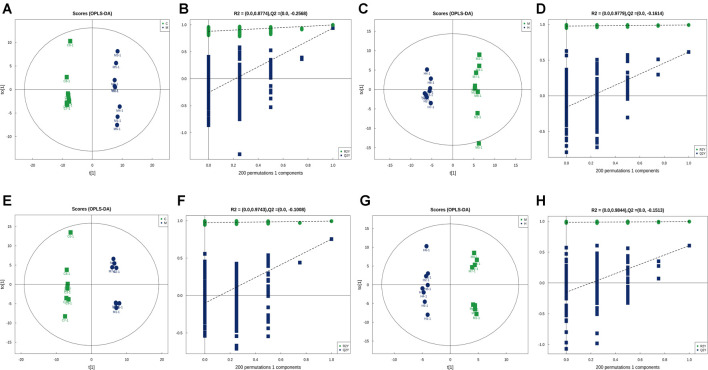
In multivariate statistical analysis, the OPLS-DA label **(A)** and permutation **(B)** of the control group vs. model group and the OPLS-DA label **(C)** and permutation **(D)** of the model vs. H-RGG group for negative ion mode; and the OPLS-DA label **(E)** and permutation **(F)** of the control group vs. model group and the OPLS-DA label **(G)** and permutation **(H)** of the model vs. H-RGG group for positive ion mode.

To investigate the mechanism of action of RGGs against liver fibrosis, metabolomic profiling of serum using a UHPLC-Q/TOF-MS system with multivariate statistical analysis was performed to determine changes in the metabolome in different groups. From the representative total ion current chromatograms, 309 metabolites were obtained in both the positive and negative modes, including 121 undefined metabolites, 65 organic acids and derivatives, 56 lipids and lipid-like molecules and others (Figure 6A). Then, the QC samples were clustered in the line of the PCA score plot, and the instrument was stable during LC–MS analysis. Supervised PLS-DA analyses were performed to discriminate between the differential metabolites of each pair of groups (Figures 6B,C; Figure 7).

The variable importance for projection (VIP) reflects the importance of variables; hence, based on the VIP value, fold change (FC) value and *p* value, the different metabolites from the different groups were obtained. Briefly, using VIP >1.0, FC > 1.2 or FC < 0.833 and *p* < 0.05 as the threshold, 49 and 23 differential metabolites were obtained for the control group vs model group and the model group vs. RGG group for negative ions, and 16 common metabolites were significantly regulated in comparison with the control, model and RGG groups (Figure 6D); for positive ions, 20 and 12 differential metabolites were obtained for the control group vs model group and the model group vs RGG group for negative ions, and only 4 common metabolites were significantly regulated in comparison with the control, model and RGG groups (Figure 6E; Table 2).

**TABLE 2:** Penitential biomarkers associated with RGG treatment of liver fibrosis in rat serum in positive mode and negative mode.

Mode	Metabolites	tR/s	Mass (m/z)	FC_M_	FC_R_	Control vs. Model	Model vs. RGG
Negative	D(-)-Beta-hydroxy butyric acid	234.52	103.04	0.32	0.55	Up^b^	Up^d^
	11-Keto-.beta.-boswellic acid	99.05	469.33	0.13	2.13	Up^b^	Down^d^
	1-Stearoyl-sn-glycerol 3-phosphocholine	187.22	522.35	2.97	0.58	Down^b^	Up^d^
	2E-Eicosenoic acid	38.39	309.28	1.64	0.67	Down^b^	Up^d^
	Taurochenodeoxycholate	147.04	498.29	0.15	2.91	Up^b^	Down^d^
	Thromboxane B2	171.40	362.23	0.35	1.88	Up^b^	Down^d^
	all *cis*-(6,9,12)-Linolenic acid	45.49	277.22	1.49	0.64	Down^a^	Up^d^
	Thymine	98.38	125.03	0.67	1.48	Up^b^	Down^d^
	Maslinic Acid	95.55	471.35	0.07	2.03	Up^b^	Down^d^
	Citraconic acid	442.79	129.02	0.28	1.50	Up^b^	Down^d^
	Cysteine-S-sulfate	316.98	199.97	0.66	1.28	Up^b^	Down^d^
	Deoxycholic acid	133.65	391.28	0.20	1.71	Up^b^	Down^d^
	Thymidine	98.21	241.08	0.80	1.26	Up^a^	Down^d^
	p-Cresol	25.12	107.05	0.13	0.59	Up^b^	Up^c^
	L-Valine	303.90	116.07	0.65	0.76	Up^a^	Up^c^
	Adynerin	515.30	53.15	0.06	5.69	Up^a^	Down^c^
Positive	1-Palmitoyllysophosphatidylcholine	186.46	538.38	2.48	0.61	Down^b^	Up^d^
	1-Myristoyl-sn-glycero-3-phosphocholine	196.52	468.31	1.34	1.31	Down^b^	Down^c^
	DL-Phenylalanine	229.56	207.11	1.36	0.82	Down^b^	Up^c^
	Trimethylamine N-oxide	325.27	76.08	0.34	2.63	Up^a^	Down^c^

The FC_M_ and FC_R_ values represent the fold change of the model group compared to the control group or RGG group, respectively.^a^ *p < 0.05*, ^*b*^ *p < 0.01* compared with the control group; ^c^ *p < 0.05*, ^*d*^ *p < 0.01* compared with the model group.

Finally, the KEGG pathway of all common metabolites was evaluated using MetaboAnalyst 5.0 (https://www.metaboanalyst.ca/). Figure 6F shows that the differential metabolites were mainly enriched in pyrimidine metabolism, phenylalanine, tyrosine and tryptophan biosynthesis, phenylalanine metabolism, primary bile acid biosynthesis and aminoacyl-tRNA biosynthesis. These pathways might denote their potential as the targeted pathways of RGGs against liver fibrosis induced by CCl_4_.

## Discussion

As a frequent pathological feature of chronic hepatic insults, liver fibrosis seriously affects human health and causes significant morbidity and mortality worldwide (Puche et al., 2013). Due to their complex pathological features and pathogeny, promising chemical drugs have not been found until now. In recent years, TCMs, as an alternative therapy, have demonstrated clinical efficiency by attenuating the primary disease, inhibiting myofibroblast activation, advancing the apoptosis or reversion of activated stellate cells and reducing matrix degradation (Xiong et al., 2020). However, it is a great challenge to clarify the mechanism of action of TCM because of the complex compounds and synergistic mechanisms (Tan et al., 2012). Network pharmacology has attracted the attention of many colleagues, and it is more effective to analyze drug combinations and explain their potential mechanism of action by establishing a “compound-protein/gene-disease” network in a high-throughput manner, especially for TCM preparations (Zhang et al., 2019). Metabolomics technology has been used to bridge the gap between TCM and molecular pharmacology by determining thousands of metabolites in living systems (Wang et al., 2005), and the differential metabolites of drug groups and model groups have been widely applied in exploring metabolic pathways. Currently, integrated network pharmacology and metabolomics have been widely used to investigate the mechanism of action of TCMs, such as the antidepressant mechanisms of Xiaoyaosan (Liu et al., 2021) and detoxification mechanisms of Yunnanbaiyao (Ren et al., 2020). Several successful metabolomics or network pharmacology tests on the anti-liver fibrosis mechanism of TCMs have been reported (Chen et al., 2019; Xiong et al., 2020; Zhang et al., 2021).

RGG treatment has been widely used to treat liver fibrosis with good efficiency in the clinic. To ensure the quality and safety of this preparation, we determined the contents of active compounds and evaluated the acute toxicity of RGGs. The results showed that at the given contents and doses, RGGs were safe and presented anti-liver fibrosis activity. Further studies showed that RGGs protected liver function and improved pathology by decreasing the CCl_4_-induced increase in ALT and AST levels in rats and alleviated or even reversed liver fibrosis in rats by decreasing the serum levels of fibrosis markers caused by CCl_4_, including HA, LN, PC III, HYP, CoII-V, and α-SMA. In addition, RGG treatment reduced the oxidant stress and inflammatory response of rats compared with the model group. These *in vivo* tests proved the clinical use of RGGs for treating liver fibrosis, and RGGs had a good effect on liver fibrosis, especially at high doses.

Simplified network pharmacology was used to understand the relationship between RGGs and liver fibrosis to explore their mechanism. A PPI network showed that as hub and core targets, SRC, PIK3R1, STAT3 and AKT1 were potential anti-liver fibrosis targets of RGGs. SRC may play a role in the regulation of embryonic development and cell growth. Src kinase regulates the dynamic interaction between resident fibroblasts and the ECM by regulating integrin and focal adhesion proteins to regulate cell proliferation and migration (Li et al., 2020) and is considered to be the key factor for treating fibrotic diseases. A Src family kinase inhibitor, THU-081-saracatinib, has been proven to decrease liver fibrosis (Seo et al., 2019). Meanwhile, much evidence has confirmed that PIK3R1, STAT3 and AKT1 can regulate crucial cellular processes, including proliferation, survival, growth, autophagy, and metabolism, and improve liver fibrosis (Nunes-Santos et al., 2019). Hence, we thought that RGGs presented anti-liver fibrosis activity by regulating the expression of SRC, PIK3R1, STAT3, AKT1 and other core targets.

Subsequently, we screened the active compounds using a target compound network, and kaempferol, isorhamnetin, quercetin, and luteolin with multiple targets in liver fibrosis were found. These compounds are widely distributed in nature and present a wide range of pharmacological activities, such as antioxidant, anticancer, anti-inflammatory, antiatherogenic, and neuroprotective properties. Result showed that kaempferol, morin, quercetin, and luteolin could be used as active compounds in RGGs, contributing to their anti-liver fibrosis activity, and they all presented the inhibitory effect on LX-02 cells activated by TGF with a concentration-dependent manner (Figure 3). Previous studies have indicated that quercetin ameliorates CCl_4_-induced liver fibrosis via the HMGB1-TLR2/4-NF-κB, NF-кB/IкBα, p38 MAPK, and Bcl-2/Bax signaling pathways (Li et al., 2016b; Wang et al., 2017) and that luteolin can reverse the liver fibrosis in mice induced by CCl_4_ (Domitrović et al., 2009). Kaempferol and isorhamnetin have also been proven to affect fibrosis, and the pharmacological effects of isorhamnetin are related to the regulation of NF-κB, PI3K/AKT, MAPK and other signaling pathways (Li et al., 2016; Gong et al., 2020; Kim et al., 2015). The high affinity between active compounds (kaempferol, quercetin) and targets (SRC and AKT1) were verified using molecular docking, which has been widely used to study the interaction between small molecule and target protein via visualizing the binding mode for the structure-based drug discovery (Zhou et al., 2021). Reports showed that quercetin and kaempferol restrained the PI3K-Akt signal pathway by inhibiting the expression of p-Akt1 (Lu et al., 2018; Imran et al., 2019), and the latter compound also suppressed the expression of Src contributing to its anti-inflammatory activity (Imran et al., 2019).

KEGG pathway analysis showed that these potential targets were mainly enriched in pathways in cancer, the PI3K-AKT signaling pathway, and the MAPK signaling pathway. The PI3K-Akt signaling pathway controls various kinds of cellular processes, including autophagy, survival and differentiation (Wang et al., 2020b). Currently, this pathway has become a target in the antifibrotic approach in the liver. When PI3K is activated, Akt is phosphorylated, and then, the inhibition of Akt phosphorylation suppresses HSC activation, ameliorates collagen and ECM deposition and causes HSC apoptosis (El-Mihi et al., 2017). Considering that the PI3K-Akt signaling pathway participates in the development of fibrosis, we hypothesized that RGGs exert anti-liver fibrosis activity by inhibiting this signaling pathway. It was proved in PCR test that RGG inhibited the activation of PI3K-Akt pathway induced by CCl_4_ to block the progress of liver fibrosis with a dose-dependent manner.

From Table 2, the penitential biomarkers associated with RGG treatment of liver fibrosis in rat serum were found, and some of them participated into the liver fibrosis. The accumulation of beta-hydroxy butyric acid could trigger severe steatohepatitis and then promote liver fibrosis progression (Liao et al., 2021), however, the lack of this substance will result in inflammation response and liver injury indirectly PI3K-Akt pathway (Miyauchi et al., 2019). Hence, we hypothesized that RGG regulated the beta-hydroxy butyric acid production to prevent liver fibrosis *via* PI3K-Akt pathway. The primary bile acid metabolism and bile acid biosynthesis played an important role to keep liver function and liver fibrosis, the serum concentration of total bile acids increases with the degree of decompensation of liver cirrhosis, such as deoxycholic acid (Xiong et al., 2020; Sauerbruch et al., 2021). In addition, pyrimidine metabolism encompasses enzymes involved in the synthesis, degradation, salvage, interconversion and transport of these molecules, such as RNA and DNA synthesis, and formation of UDP sugars for glycosylation of proteins and lipids, and pyrimidines can be degraded to simple metabolites to become sources of carbon and nitrogen. It also participates in mitochondrial dynamics and lipid oxidants and can be regulated by the MAPK 129 cascade (Erk1/2) signaling pathway and other pathways (Garavito et al., 2015; Miret-Casals et al., 2018). The connection between pyrimidine metabolism and mitochondrial morphology is conserved across species and cell types, and mitochondrial elongation represents an adaptive response against the depletion of pyrimidine pools and confers protection against induced apoptosis (Garavito et al., 2015). In addition, the abnormal increase of phenylalanine in patients with liver failure indicates an increased risk of hepatic encephalopathy and other liver fibrosis (Huang et al., 2021), and liver injury was correlated with plasma levels of thromboxane B2 (Nanji et al., 1993). RGG could regulate the content of DL-phenylalanine, thromboxane B2 and other metabolites. These results indicated that RGG presented the anti-liver fibrosis activity via PI3K-Akt pathway and others to regulate pyrimidine metabolism, phenylalanine, tyrosine and tryptophan biosynthesis, phenylalanine metabolism, and primary bile acid biosynthesis, and then the mitochondrial structural and function of liver cells may be improved.

Hence, we thought that RGGs inhibited the activation of the PI3K-Akt signaling pathway in rats induced by CCl_4_, regulated pyrimidine metabolism, improved oxidative stress and the inflammatory response by regulating mitochondrial morphology, and alleviated liver fibrosis.

## Conclusion

In the present study, we investigated the contents of active compounds in RGGs using HPLC and evaluated their acute toxicity. RGG treatment had a significant therapeutic effect against rats induced by CCl_4_ in a dose-dependent manner. it inhibited the activation of the PI3K-Akt signaling pathway; subsequently, pyrimidine metabolism was regulated, and oxidative stress and the inflammatory response were improved to alleviate liver fibrosis. Quercetin, and luteolin were active compounds. This paper indicated that RGGs presented anti-liver fibrosis activity through multiple pathways, multiple levels and multiple targets, and will lay the foundation for the clinical promotion and application of RGGs.

## Data Availability

The original contributions presented in the study are included in the article/Supplementary Material, further inquiries can be directed to the corresponding author.

## Glossary

ALT: Alanine transaminase
AST: Aspartate transaminase
CAT: Catalase
CCl4: Carbon tetrachloride
ColIV: Type IV collagen
DL: Drug-likeness
DMEM: Dulbecco’s modified eagle medium
GO: Gene ontology
FBS: Fetal bovine serum
HA: Ayaluronic acid
H&E: Hematoxylin and eosin
HSCs: Hepatic stellate cells
HYP: Hydroxyproline
IL-1β: Interleukin-1β
KEGG: Kyoto Encyclopedia of Genes and Genomes
LN: Laminin
MDA: Malondialdehyde
NH4AC: Ammonium acetate
NH4F: Ammonium fluoride
NH4OH: Ammonium hydroxide
OB: Oral bioavailability
OPLS-DA: Orthogonal partial least-squares discriminant analysis
PCA: Principal component analysis
PC III: Type III procollagen
PCR: Polymerase chain reaction
PI3K-AKT: Phosphatidylinositol 3 kinase-protein kinase B
PPI: Protein-protein interaction
RGG: Ruangan granules
α-SMA: α-Smooth muscle actin
SOD: Superoxide dismutase
TCM: Traditional Chinese medicines
TGF-β: Transforming growth factor-β
TNF-α: Tumor necrosis factor-α
UPLC: Ultra-high performance liquid chromatography.
