# Supplementary material for: Anti-Liver Fibrosis Activity and the Potential Mode of Action of Ruangan Granules: Integrated Network Pharmacology and Metabolomics
Source: Front Pharmacol. 2022 Jan 14;12:754807. doi: 10.3389/fphar.2021.754807 (PMC8805709; doi:10.3389/fphar.2021.754807)
Supplement: Supplementary file 1 [file DataSheet1.doc]

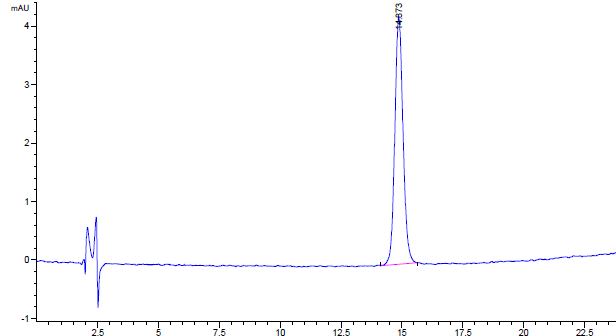
 a
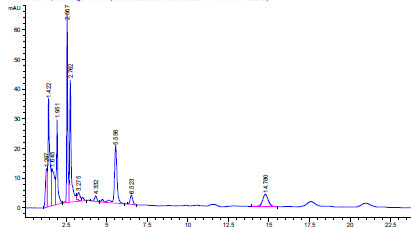
 b


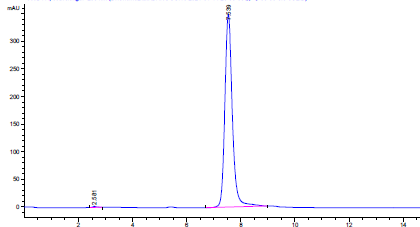
c
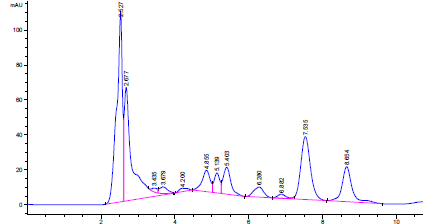
d


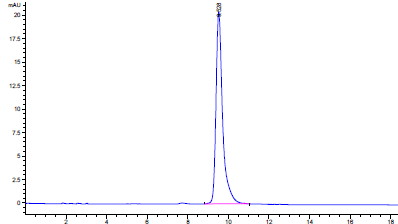
 e
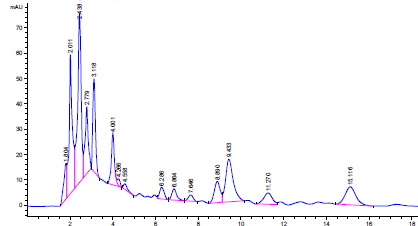
f


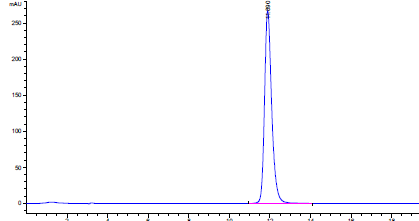
g
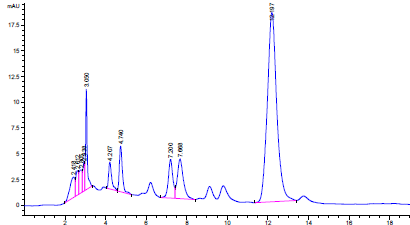
h


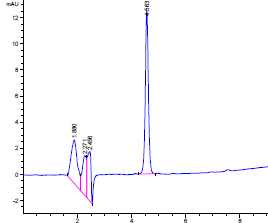
i
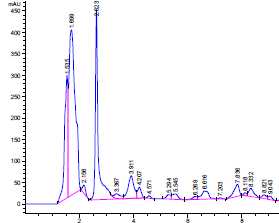
j


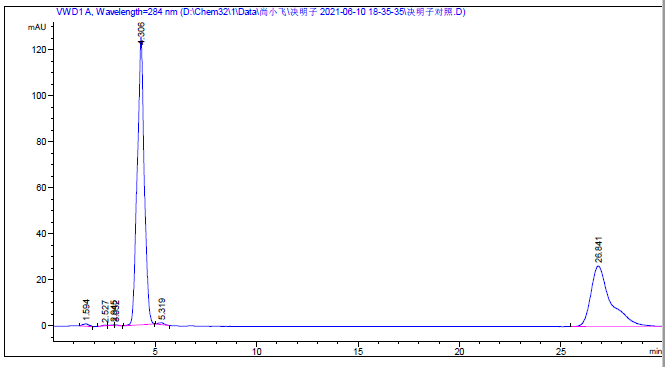
k
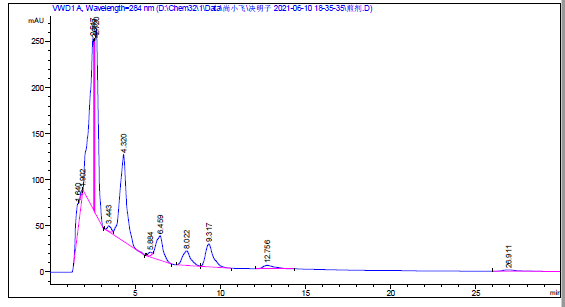
l

Figure S1. The UPLC chromatograms for determination the contents of standard and RGG for ferulic acid (a, b), paeoniflorin (c, d), salvianolic acid B (e, f), rosmarinic acid (g, h), calycosin (i, j), rhein for 4.31 min and aurantioobtusin for 26.96 min (k, l)


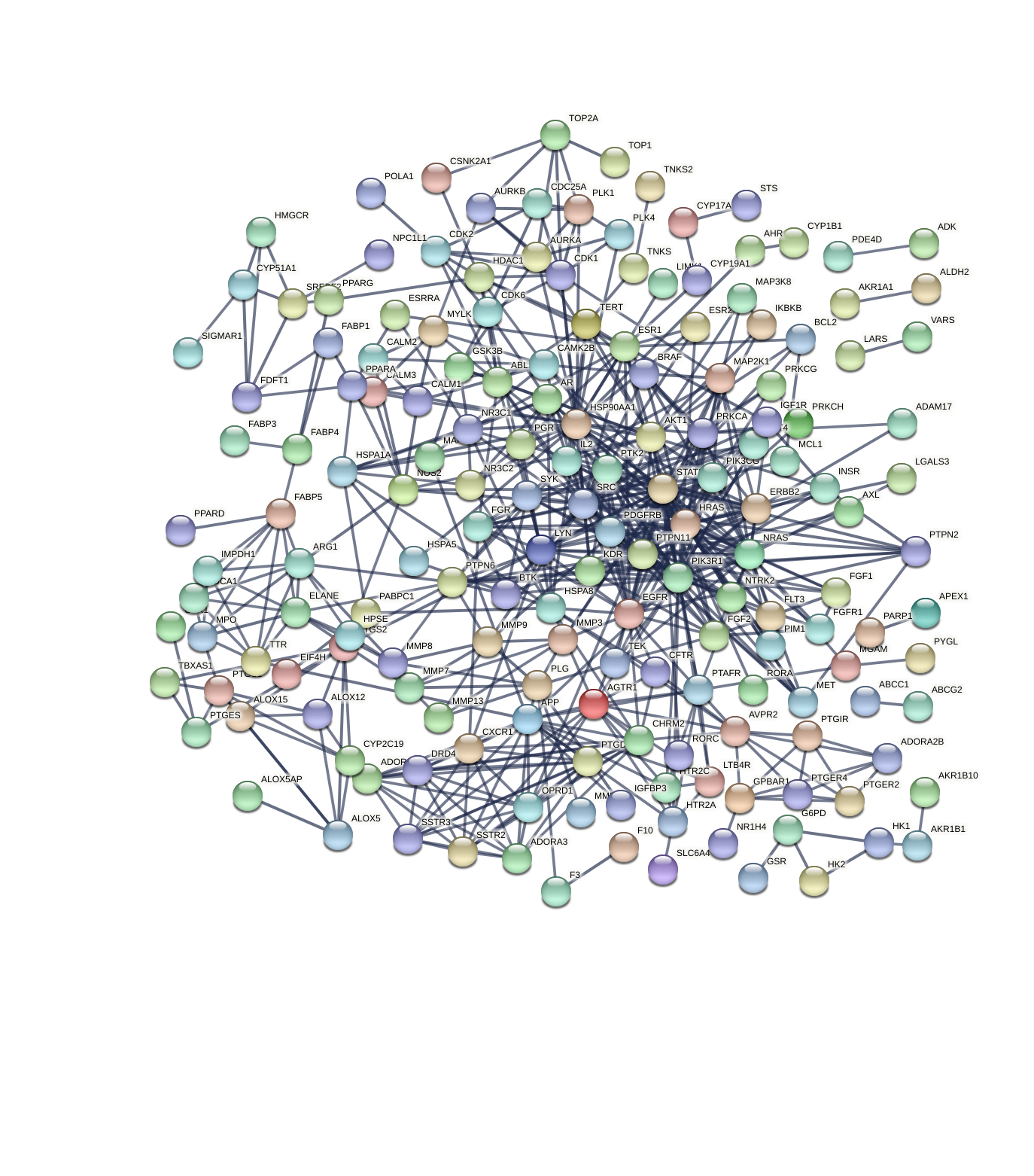


Figure S2. The protein-protein network of common targets between liver fibrosis and RGG.


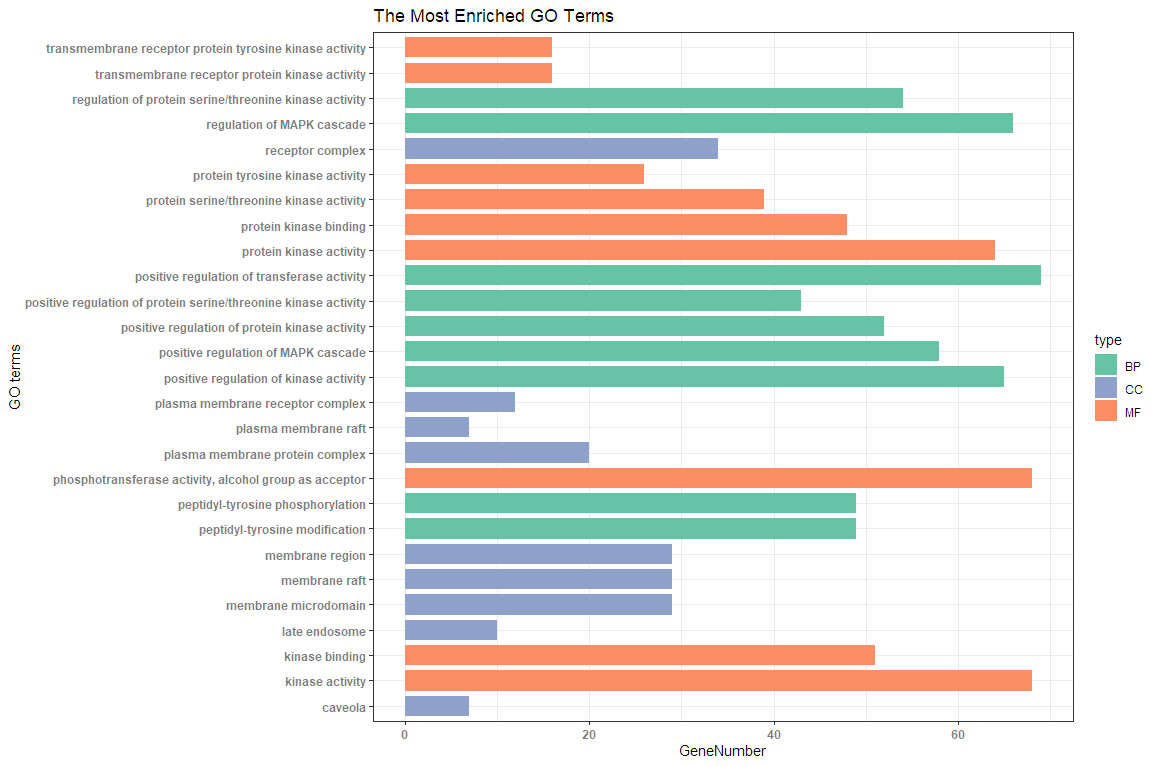


Figure S3. GO analysis of RGG against liver fibrosis.
